# Mechanisms and Engineering Research on the Processing, Storage, and Preservation of Fresh Food

**DOI:** 10.3390/foods12244402

**Published:** 2023-12-07

**Authors:** Min Zhang, Hongwei Xiao

**Affiliations:** 1 State Key Laboratory of Food Science and Technology, Jiangnan University, Wuxi 214122, China; 2 College of Engineering, China Agricultural University, Beijing 100083, China; xhwcaugxy@163.com

The past few years have witnessed a significant increase in research and development activities related to advances in the processing, storage, and preservation of fresh food. This Special Issue of *Foods* aims to highlight a set of original research and review articles on the current status, challenges, and future directions of the processing, storage, and preservation of fresh food. We sincerely appreciate the active support from *Foods*’ publishers and the kind invitation extended to us to serve as Guest Editors for this Special Issue.

Following an excellent response to our call for papers, we peer-reviewed a number of papers and selected 19—including 17 research articles and 2 review articles—to appear in this Special Issue. This Special Issue covers a wide range of fresh food topics as well as a variety of materials and technologies used in the field. These papers examine a variety of fresh foods ranging from fish and aquaculture (Chinese mitten crab, crucian carp, Yesso scallops), meat products (pig carcasses, chicken meat, pork mince), fruit and vegetables (fresh-cut kiwifruit, walnut, *Pleurotus eryngii*, fresh pepper fruit), processed food (pre-prepared fried rice, processed cheese) and aflatoxin in fresh food. Relatively new technologies for the preservation, processing and detection of fresh food during storage and transportation are presented in this Special Issue, including various plant essential oil components, hydrogen-rich water and slightly acidic electrolyzed water, high-quality drying, 3D and 4D functional printing, intelligent food packaging indicators for detecting freshness, and a novel detection technique for aflatoxin M1.

In the future, emerging technologies for the processing, storage, and preservation of fresh food with environmentally friendly, cost-effective, energy-saving, high-quality, intelligent-detection, and regulation characteristics will be pursued vigorously for the sustainable development of fresh food in China as well as globally. We hope that this Special Issue will further encourage research interest, engineering practice, and international communication in the field of fresh food.

We believe that all the articles published in this Special Issue will provide valuable information to scientists, engineers, researchers, and manufactures throughout the world, working in academia as well as in industry in the processing, storage, and preservation of fresh food.

